# Morphological and genomic characterisation of the *Schistosoma* hybrid infecting humans in Europe reveals admixture between *Schistosoma haematobium* and *Schistosoma bovis*

**DOI:** 10.1371/journal.pntd.0010062

**Published:** 2021-12-23

**Authors:** Julien Kincaid-Smith, Alan Tracey, Ronaldo de Carvalho Augusto, Ingo Bulla, Nancy Holroyd, Anne Rognon, Olivier Rey, Cristian Chaparro, Ana Oleaga, Santiago Mas-Coma, Jean-François Allienne, Christoph Grunau, Matthew Berriman, Jérôme Boissier, Eve Toulza

**Affiliations:** 1 IHPE, Univ Perpignan Via Domitia, CNRS, IFREMER, Univ Montpellier, Perpignan, France; 2 CBGP, IRD, CIRAD, INRAE, Institut Agro, Univ Montpellier, Montpellier, France; 3 Wellcome Sanger Institute, Wellcome Genome Campus, Hinxton, United Kingdom; 4 Institute for Mathematics and Informatics, University of Greifswald, Greifswald, Germany; 5 Department of Computer Science, ETH Zürich, Zürich, Switzerland; 6 Parasitology Laboratory, Instituto de Recursos Naturales y Agrobiología de Salamanca (IRNASA, CSIC), Cordel de Merinas, Spain; 7 Departamento de Parasitologia, Facultad de Farmacia, Universidad de Valencia, Burjassot, Valencia, Spain; Natural History Museum, UNITED KINGDOM

## Abstract

Schistosomes cause schistosomiasis, the world’s second most important parasitic disease after malaria in terms of public health and social-economic impacts. A peculiar feature of these dioecious parasites is their ability to produce viable and fertile hybrid offspring. Originally only present in the tropics, schistosomiasis is now also endemic in southern Europe. Based on the analysis of two genetic markers the European schistosomes had previously been identified as hybrids between the livestock- and the human-infective species *Schistosoma bovis* and *Schistosoma haematobium*, respectively. Here, using PacBio long-read sequencing technology we performed genome assembly improvement and annotation of *S*. *bovis*, one of the parental species for which no satisfactory genome assembly was available. We then describe the whole genome introgression levels of the hybrid schistosomes, their morphometric parameters (eggs and adult worms) and their compatibility with two European snail strains used as vectors (*Bulinus truncatus* and *Planorbarius metidjensis*). Schistosome-snail compatibility is a key parameter for the parasites life cycle progression, and thus the capability of the parasite to establish in a given area. Our results show that this *Schistosoma* hybrid is strongly introgressed genetically, composed of 77% *S*. *haematobium* and 23% *S*. *bovis* origin. This genomic admixture suggests an ancient hybridization event and subsequent backcrosses with the human-specific species, *S*. *haematobium*, before its introduction in Corsica. We also show that egg morphology (commonly used as a species diagnostic) does not allow for accurate hybrid identification while genetic tests do.

## Author summary

In 2013, schistosomiasis reached Southern Europe. Since then, endemic infections were recurrently identified clearly indicating that the parasite has settled and established locally. Using two molecular markers, we had previously demonstrated that the parasite was a hybrid between *Schistosoma haematobium* and *Schistosoma bovis*, two species known to infect humans and livestock, respectively. Nevertheless, this method has very low resolution and is not informative for determining the origins and the mechanisms of hybridization events, e.g. if the hybrid had been recently generated in Corsica or long before it’s introduction. The genome-wide sequencing approach, used in this study, allowed us to reveal genomic admixture suggesting that these hybrids are the result of ancient crossing events between *S*. *haematobium* and *S*. *bovis*, with subsequent backcrossing with *S*. *haematobium*. This introgression has prevented compatibility with *P*. *metidjensis* snail vectors, which are specific to *S*. *bovis*. Additionally, whether in Africa or in Europe, a clear discrepancy exists between the egg shape usually used for species identification and the genomic composition of schistosomes. Therefore, egg shape cannot be used as a good indicator for hybrid detection. Knowing the phenotypic traits and the genetic features of such hybrids may have important implications in terms of diagnostics and disease management either through vector control strategies or treatment of patients.

## Introduction

Schistosomes are dioecious parasitic flatworms, responsible for the major Neglected Tropical Disease (NTD) schistosomiasis. The epidemiological statistics associated with this disease are sobering: 800 million people are at risk in 78 countries, mostly concentrated in sub-Saharan Africa; 230 million are infected and the disease causes more than 200,000 deaths each year as well as between 1.7 and 4.5 million Disability Adjusted Life Years (DALYs) [1]. The most exposed groups are children and young adults who have activities linked to contaminated freshwater environments. In addition to humans, schistosomiasis severely impacts livestock in Africa and Asia with over 165 million animals estimated to be infected [2].

The parasites have a complex life cycle that includes passage through specific freshwater snail intermediate hosts (hereafter termed vectors) in which the parasites undergo clonal multiplication, and a final vertebrate definitive host in which, adult worms sexually reproduce. The liberation of *Schistosoma* eggs into the host tissues is the principal cause of chronic and acute morbidity [3].

Global changes, including both anthropogenic and environmental modifications, may contribute to modifications in the geographical distribution of species and expand their potential ecological niches [4,5]. Distinct species may thus acquire a new capacity to interact, hybridize and subsequently introgress their genomes by backcrossing with parental species or other hybrids, a phenomenon called “hybrid swarm” [6]. Hybridization between individuals from two previously reproductively isolated species is generally expected to result in the production of offspring less fit than the parents, sometimes non-viable or sterile. In some cases however, hybridization may lead to viable progeny that can even have a greater fitness than parental species, a genetic effect known as hybrid vigour or heterosis that is generally observed during early generations [7]. These advantageous combinations of parental genes in offspring may enable progeny to adapt to new environments as potentially exemplified by the recent outbreak of schistosomiasis in Southern Europe (Corsica, France) [8].

Hybridization events involving parasites of humans are not scarce and are a real concern in terms of parasite transmission, epidemiology and disease [9]. Natural hybridizations between schistosomes have already been identified: (i) between different human-specific *Schistosoma* species, (ii) between different animal-specific *Schistosoma* species, (iii) and between human-specific and animal-specific *Schistosoma* species [10]. These latter hybrid forms are particularly alarming because they raise the possibility of the emergence of new zoonotic parasitic strains, introducing animal reservoirs and therefore greatly hampering our ability to properly control transmission.

The precise characterization of the genetic composition and the introgression levels of hybrid populations is thus essential for a identification purpose, but is also necessary to better understand the parasite life history traits, as well as the disease dynamics and epidemiology in the field.

To this end, next generation whole-genome sequencing is now the tool of choice for a deeper insight into the genomic composition of natural hybrids. In particular, it may enable for a better understanding of these hybridization events, if they are frequent and active, rare and/or ancient together with the direction of the genetic introgression which may result in the inheritance of species specific phenotypic traits [11–13]. This genomic interrogation may also provide valuable insights into reproductive isolating barriers that are at play helping to maintain species integrities and prevent hybrid speciation [11,14,15]. Although recent studies on the absence of pre-zygotic isolation mechanisms suggest that hybridization between *S*. *haematobium* and *S*. *bovis* may be common [16], current genomic analyses of hybrids recovered from endemic areas indicate that introgression between *S*. *haematobium* and *S*. *bovis* is the result of ancient events [11,14,15]. Only a small proportion of the *S*. *bovis* genome appears to be introgressed into the genomic background of *S*. *haematobium*, with a potential adaptive significance related to host-pathogen interactions [11,15].

In this study, we aimed to fully characterizing *S*. *haematobium-bovis* hybrids that have emerged in Europe in summer 2013. We describe the European *S*. *haematobium-bovis* hybrids based on the morphology of their eggs (the disease- and diagnostic-relevant stage), and laboratory bred adult worms, as well as compatibility with two potential European snail vectors. Finally, we characterized the extent of introgression in the European natural *S*. *haematobium-bovis* hybrids at the whole genome level.

## Results

### Morphological description of the European *S*. *haematobium-bovis* hybrid eggs and adult worms

A total of 44 eggs collected from hamsters infected with the European *S*. *haematobium-bovis* hybrid were examined for morphological characterization. The length and width were measured for all 44 eggs, however, the spine length was only measured for a subset of 36 eggs, due to it not being distinctive enough to allow for accurate measurements for eight of the eggs. The eggs of the European *S*. *haematobium-bovis* hybrid showed a high variability and ranged between 73.9–170.9 μm in length (mean: 126.4 ± 22.9 standard deviation), 40.9–92.5 μm in width (mean: 60.8 ± 13.0 standard deviation) and 3.95–13.6 μm for the spine length (mean: 8.2 ± 2.1 standard deviation).

**Fig 1:**
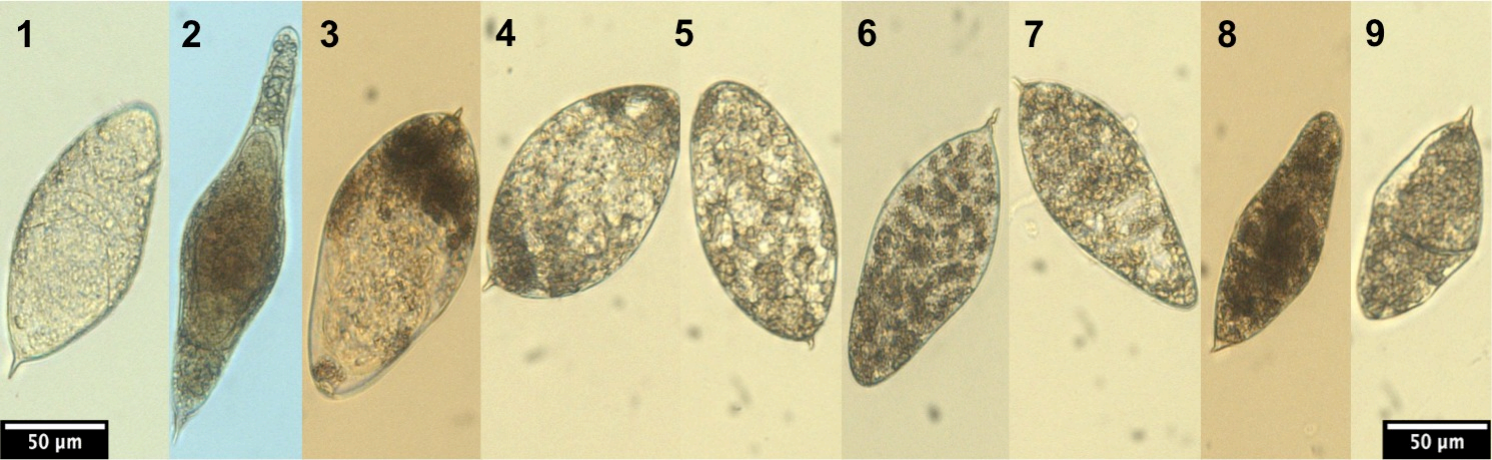
Egg morphologies of the pure parental species and the European *S*. *haematobium-bovis* hybrids. Eggs 1 and 2 show typical morphologies of *S*. *haematobium* (elliptical with a terminal spine) and *S*. *bovis* (spindle shape with a terminal spine), respectively from laboratory isolates. Eggs 3–9 show the egg morphology of the European *S*. *haematobium-bovis* hybrid schistosome. While most eggs were of a typical *S*. *haematobium* morphotype (3–7), there was a high variability in the morphologies and near 10% (4/44) of eggs were non-typical (8–9).

Most of the eggs displayed representative elliptical morphotypes and were all characterized by a terminal spine, reminiscent of *S*. *haematobium* infection in humans. Nevertheless, not all eggs had a typical *S*. *haematobium* morphotype and were in some cases similar to *S*. *bovis*-type eggs (Fig 1). In summary, egg morphology alone cannot differentiate between the hybrid and putative parental species. Nevertheless, morphological analysis together with genetic markers supports the initial conclusion that the hybrid originates from a cross between *S*. *bovis* and *S*. *haematobium*.

As with all other *Schistosoma* species strong sexual dimorphism was observed for the *S*. *haematobium-bovis* hybrids with females being held within the gynaecophoric canal of the males. The detailed morphological characteristics and morphometric measurements of the adult worms are presented in Table 1.

**Table 1:** Morphological measurements of the European male and female hybrid adult worms.

	Hybrid schistosomes from Corsica			
	Male		Female	
	Mean (±SD)	Range	Mean (±SD)	Range
**Total length (mm)**	5.3 (±1.0)	3.3–6.6	9.6 (±1.2)	8.6–14.1
**Largest body width (mm)**	0.3 (±0.07)	0.2–0.5	0.2 (±0.04)	0.1–0.3
**Oral sucker length (mm)**	0.2 (±0.01)	0.2–0.2	0.05 (±0.004)	0.04–0.06
**Oral sucker width (mm)**	0.2 (±0.006)	0.1–0.2	0.05 (±0.002)	0.05–0.06
**Ventral sucker length (mm)**	0.3 (±0.008)	0.2–0.3	0.06 (±0.001)	0.06–0.07
**Ventral sucker width (mm)**	0.3 (±0.01)	0.2–0.3	0.06 (±0.001)	0.06–0.07
**Testes number**	4.2 (±0.5)	4–5	-	-
**Ovary length (mm)**	-	-	0.3 (±0.01)	0.3–0.3
**Ovary width (mm)**	-	-	0.09 (±0.01)	0.08–0.1
**Extension of the vitellaria (mm)**	-	-	5.0 (±0.7)	4–5.9

**Fig 2:**
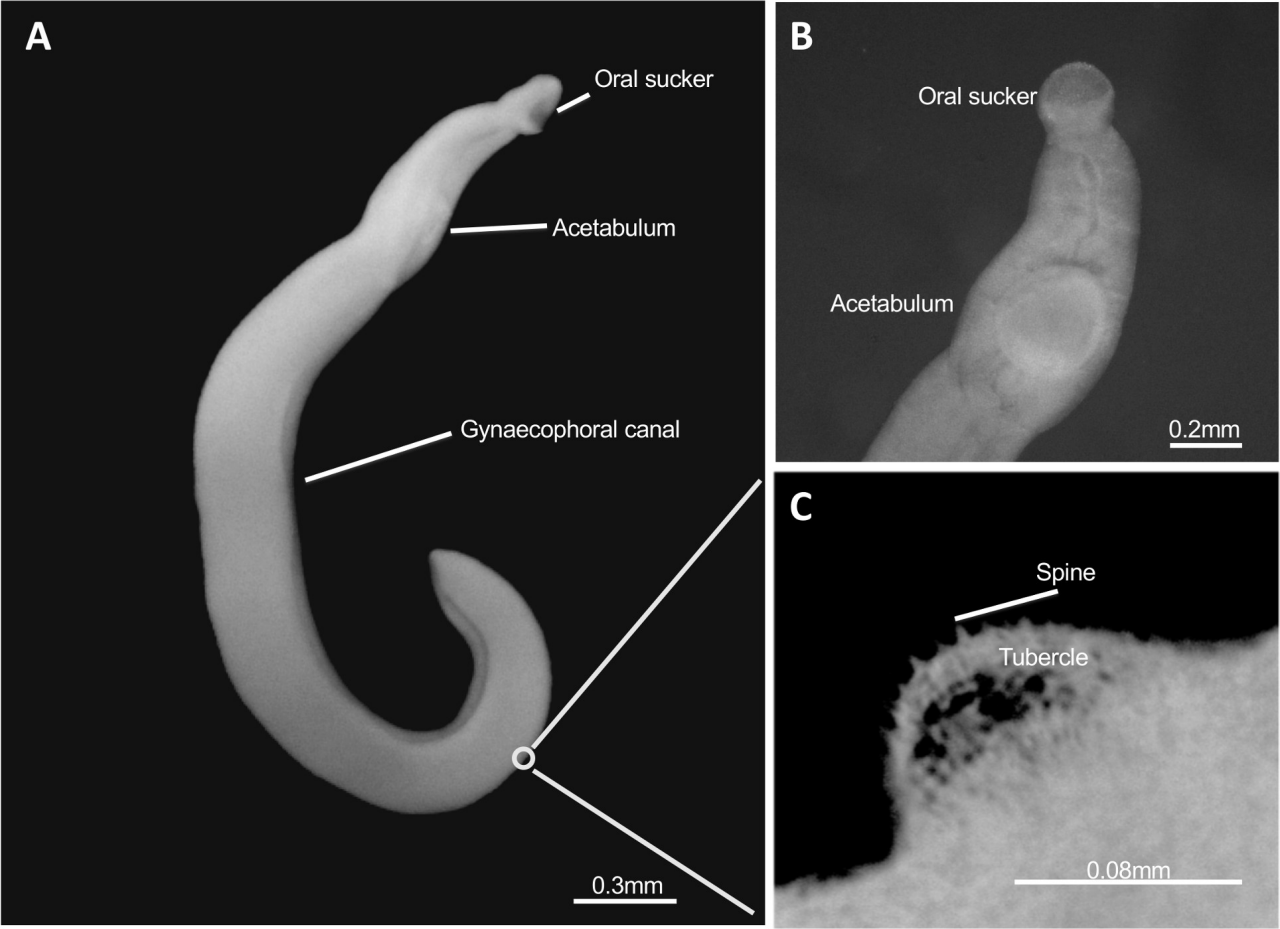
Morphological observations of the European male hybrid adult worms. (A) Whole parasite body showing the oral sucker, acetabulum (ventral sucker) and gynaecophoral canal; (B) Frontal view of the anterior region showing the oral sucker and acetabulum (ventral sucker) in detail; (C) Dorso-lateral view of the spines and tubercles on the parasite surface.

The adult *S*. *haematobium-bovis* hybrid males had an elongated body measuring, on average, 5.3 (±1.0) mm in length and 0.3 (±0.07) mm in width at largest part of the body (middle). The male body was dorso-ventrally flattened with folds in the ventral axis forming the gynaecophoral canal (Fig 2A). The anterior region was narrower than the rest of the body and presented a sub-terminal mouth formed into the oral sucker (length: 0.2±0.01 mm; width: 0.2±0.006 mm) and a robust ventral sucker or acetabulum (length: 0.3±0.008 mm; width: 0.3±0.01 mm) near the top of the gynaecophoral canal (Fig 2B). The oesophagus began at the oral sucker and was extended posteriorly to the acetabulum where it bifurcates into the gut caeca until it reunites at the posterior end of the body.

**Fig 3:**
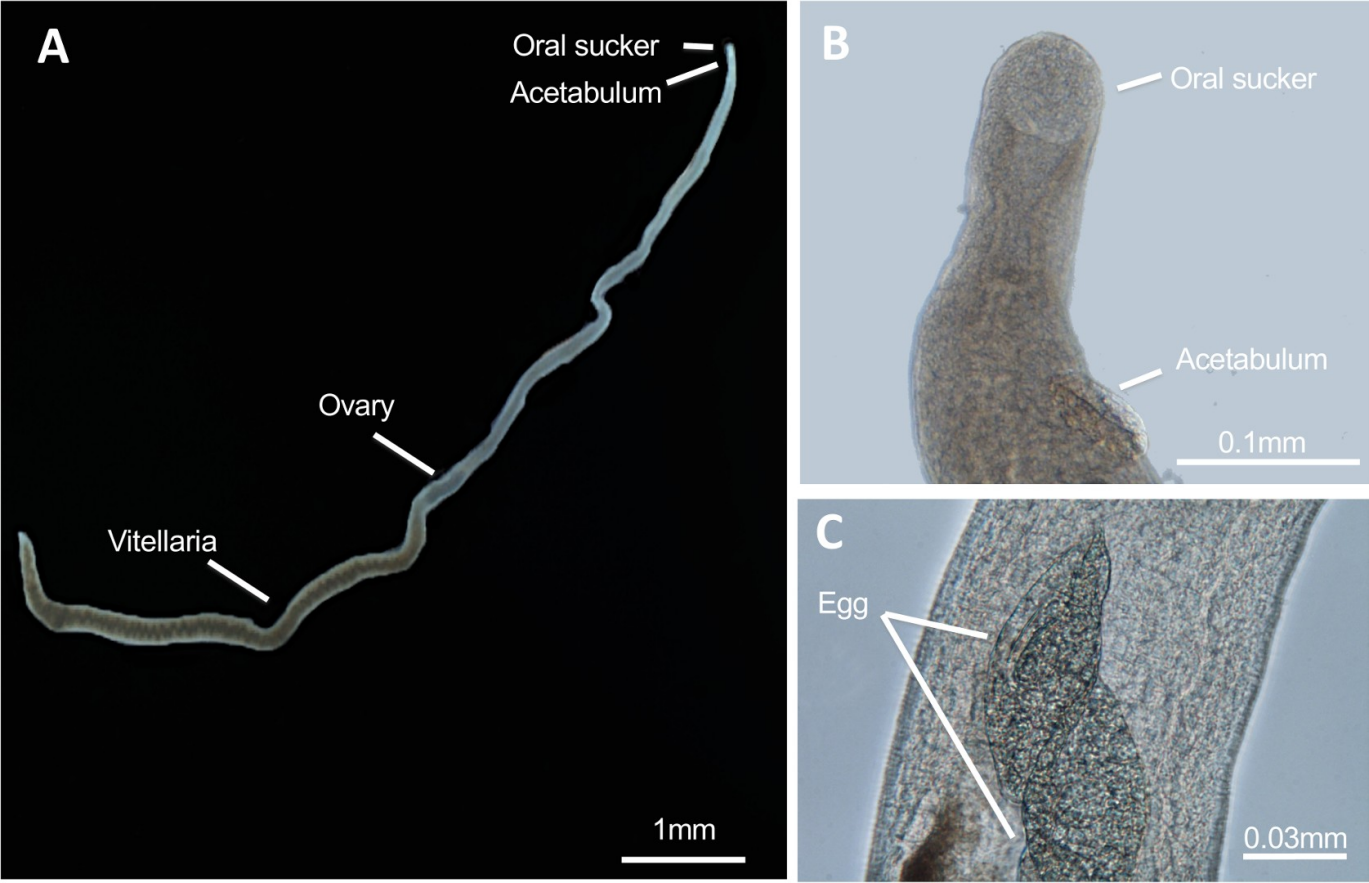
Morphological observations of the European female hybrid adult worms. (A) Lateral view of whole parasite body showing oral sucker, acetabulum (ventral sucker), ovary and vitellaria; (B) Frontal view of anterior region showing oral sucker, acetabulum (ventral sucker) in detail; (C) Dorso-lateral view of two eggs inside female uterus.

Four to five testes were observed per male, situated dorsally posterior to the ventral sucker and were round to ovoid (4.2±0.5). Posterior to the acetabulum, in the dorsal region, tubercles with a round extremity began to appear at the level of the gynaecophorial canal and occurred to all the posterior region of the body (Fig 2C). The presence of tegument projections on the tubercles where identified with several apical spines which decreased in distribution and size towards the back and sides of the male’s bodies (Fig 2C). The females were elongated and filiform measuring 9.6 (±1.2) mm in length and 0.2 (±0.04) mm in width, with the posterior half of body expanded (Fig 3A). The anterior regions of the females were smaller compared to males, had a small oral sucker (length: 0.05±0.004 mm; width: 0.05±0.002 mm) and acetabulum (length: 0.06±0.001 mm; width: 0.06±0.001 mm) (Fig 3B).

The oesophagus started near to the oral sucker and bifurcated immediately after the acetabulum. The genital pore was situated at the posterior end of the ventral sucker (dorsally) and some eggs could be observed in the uterus (Fig 3C). A single ovary measuring 0.3 (±0.01) long and 0.09 (±0.01) wide was situated in the posterior third of the female’s body (Fig 3A). The vitelline glands, also called vitellaria were extensive, occupying roughly 50% of the posterior part of the worm and extending further posteriorly than the intestine. The females’ tegument was smooth and uniform without significant projections. The posterior extremity was tapered and rounded.

### Whole genome sequencing shows introgression of *S*. *bovis* into *S*. *haematobium*

Unfortunately, we were not able to produce enough high molecular weight genomic DNA to directly proceed to long-read sequencing of individual hybrid adult worms. To better characterize the level of hybridization we therefore used Illumina short read high-throughput sequencing and alignment to parental genomes. As the previous *S*. *bovis* assembly was highly fragmented (111 328 scaffolds, N50 7kb), and another *S*. *bovis* strain from Spain was used to produce the experimental hybrids examined in this study (as below) we resequenced the *S*. *bovis* (Villar de la Yegua, Salamanca, Spain) genome using 200 worms with PacBio long reads. This produced 4,102,584 filtered subreads (48,987,175,429 bases). The genome assembly led to a new genome version of 486 scaffolds (N50: 3.1Mb) from which 14,104 protein-coding genes were identified with an average length of 18,725 bp (5.3% of genome length) (Table 2), consistent with the known characteristics of *Schistosoma* genomes [17,18].

**Table 2:** Assembly and gene prediction metrics of *S*. *bovis* from Spain.

Total assembly length (bp)	446,282,422
Number of scaffolds	486
N50 (bp)	3,109,635
Number of scaffolds ≥ N50 (L50)	38
Proportion of the genome that is coding (%)	5.27
Number of genes	14,104
Average gene length (bp)	18,725 ± 21,625
Average number of exons per gene	7.9
Average exon length (bp)	299
Average intron length (bp)	2,389
Repeat rate (%)	49.5%

Using the Benchmarking Universal Single-Copy Orthologs (BUSCO) [19] to assess the genome assembly and annotation completeness, we identified 66.9% of complete BUSCOs for our *S*. *bovis* genome (Spanish strain), compared to 63.3%, 59.7% and 62.7% for the previously sequenced *S*. *bovis* strain from Tanzania [14] and the *S*. *haematobium* v1 [17] and v2 [18] assembly, respectively using the same pipeline (Table 3). Due to the divergence of the Platyhelminth lineage and the very limited representation of Lophotrochozoa in the BUSCO protein set, it has already been shown that BUSCO underestimate completeness, particularly using its genome mode [20,21].

**Table 3:** Genome assembly and annotation completeness using BUSCO.

	*S*. *bovis* (Spanish strain, this study)	*S*. *bovis* (Tanzanian strain [14])	*S*. *haematobium* v1 (Egyptian strain [17])	*S*. *haematobium* v2 (Egyptian strain [18])
Complete BUSCOs (C)	654	619	584	613
Complete and single-copy BUSCOs (S)	586	590	577	604
Complete and duplicated BUSCOs (D)	68	29	7	9
Fragmented BUSCOs (F)	86	94	103	111
Missing BUSCOs (M)	238	265	291	254
Total BUSCO groups searched	978	978	978	978
Completeness	66.90%	63.30%	59.70%	62.70%

For the European *S*. *haematobium-bovis* hybrid, a total of 289,873,531 reads (76.5%) were mapped against the 681.2 Mb concatenated genomes of *S*. *haematobium* [17] and *S*. *bovis* (this study), representing 42X mean coverage (Table 4). We also sequenced F1 males from experimental first generation cross between male *S*. *haematobium* (Cameroon) *x* female *S*. *bovis* (Spain) as a control. A total of 4,910,354 reads (92.3%) were mapped against a concatenate of the *S*. *haematobium* and *S*. *bovis* genomes (Table 4).

**Table 4:** Summary of the introgression level analysis of the experimental F1 hybrids (control) and the natural European hybrid strain recovered in Corsica.

	Experimental F1 genome (males)	European hybrid genome	
		Males	Females
**Mapped reads (% of total number)**	4,910,354 (92.3%)	150,994,161 (80.8%)	138,879,370 (72.2%)
**Reads mapped against *S*. *haematobium* genome**	2,417,196 (49.2%)	116,052,836 (76.9%)	106,969,327 (77%)
**Reads mapped against *S*. *bovis* genome**	2,493,158 (50.8%)	34,941,325 (23.1%)	31,910,043 (23%)
**Reads mapped against *S*. *haematobium* mitochondrion**	168 (1.7%)	50,436 (2.2%)	33,454 (2.2%)
**Reads mapped against *S*. *bovis* mitochondrion**	9,825 (98.3%)	2,229,096 (97.8%)	1,455,815 (97.8%)

The mapping of the F1 reads to the *S*. *haematobium* and *S*. *bovis* genomes was of ~50% on each of the parental species genomes (Table 4). This control was crucial to validate our analytical pipeline and thus validate the results obtained for the natural European hybrid schistosomes. Moreover, 98.3% of reads of mitochondrial origin, aligned to the mitochondrial genome of *S*. *bovis*, which is consistent with the maternal inheritance of mitochondrial genome within *Schistosoma* species (Table 4).

Interestingly, the mapping of the European hybrid reads against the *S*. *haematobium* and *S*. *bovis* reference genomes revealed admixture between the parental genomes with a proportion of 76.9% of sequences mapping to the *S*. *haematobium*, and 23.1% mapping to the *S*. *bovis* genomes for both male and female parasites (Table 4). Alignment to the mitochondrial genomes of both parents also showed results concordant with the previous Sanger sequencing data for this marker, with 97.8% of reads being mapped to the *S*. *bovis* mitochondrial genome, and 2.2% to the *S*. *haematobium* mitochondrial genome [8]. To assess the divergence level between the nuclear genomes of the two “pure” species, we identified homologous regions between *S*. *haematobium* and *S*. *bovis* using CACTUS [22]. A total of 234.5 Mb sequences aligned between the parental species, representing 64% of the *S*. *haematobium* genome length. The mean similarity of these shared sequences was 95.9% compared to ~99% and ~90% for commonly used phylogenetic markers such as ITS and cox1, respectively. The mean similarity between *S*. *haematobium* mitochondria (GenBank accession number DQ157222.2) and *S*. *bovis* mitochondria (Contig 00439F of our assembly) was 82.1%.

Besides being of fundamental interest for the evolutionary biology of the parasite, this finding could also have immediate consequences for parasite control. One of the few phenotypic features that are of relevance for infection success and for which the genetic basis is known is resistance to Oxamniquine (OXA). In *S*. *mansoni* the mutations that confer resistance occur in the SmSULT-OR gene (Smp_089320), encoding a sulfotransferase that is required for drug activation, are p.E142del and p.C35R. [23,24]. In *S*. *haematobium* the drug is not efficient due to a F39 Sm > Y54 Sh substitution [24]. We reasoned that if the corresponding S. *bovis* allele would have introgressed into the hybrid it would follow the *S*. *mansoni* trait making the hybrid more sensitive to OXA. We have undertaken reciprocal homology searches with Smp_089320 against the *S*. *haematobium* and the new *S*. *bovis* genome to identify orthologues. Orthologues exist in both genomes and both possess the F->Y Sh54 and no mutations in C Sm35 or L Sm256 or deletion in E Sm142.

### The European hybrid is compatible with the natural intermediate snail hosts of *S*. *haematobium*, *B*. *truncatus* but not with *P*. *metidjensis* which is also a host of *S*. *bovis*

The presence of 23% of *S*. *bovis* genetic material in the hybrid genome raises the possibility that the hybrid is capable of infecting specific vector snails uniquely compatible with *S*. *bovis*. To test this hypothesis we exposed *P*. *metidjensis*, to miracidia of the European hybrid found in Corsica. The prevalence in its natural host, *B*. *truncatus* from Corsica was 24% (9 infected snails out of 37) whereas, no *P*. *metidjensis* became infected (0 infected snails out of 29 alive).

## Discussion

The emergence of infectious diseases are currently among the greatest concerns of our changing world and has strong outreach effects for society. Besides the important impacts that global changes may have on the spread and transmission of tropical infectious diseases in higher latitudes, other phenomenon may combine and act as driving forces promoting the emergence of novel disease in unsuspected areas. The importance and the frequency of hybridization in infectious agents are certainly underestimated, and very little attention has been given so far to the role of genetic introgression on infectious disease emergence, spread and control [25]. In the genus *Schistosoma*, several reports have revealed that inter-species hybrids are frequent and are a real concern for human health [9,10]. In particular hybridization between *S*. *haematobium* and *S*. *bovis* have now been identified with molecular tools in Senegal, Niger, Benin, Mali, Cote D’Ivoire and also Malawi [26–30]. To date genomic evidence indicates that although only a small proportion of *S*. *bovis* seems to have introgressed in the genomic background of *S*. *haematobium* (i.e. 3–8%), introgression from *S*. *bovis* is widespread across *S*. *haematobium* populations in endemic areas. The signatures of introgression observed indicate ancient and unidirectional events with a potential adaptive significance related to host-pathogen interactions [11,14,15]. Nevertheless, it is the first time that a hybrid schistosome has been involved in a large-scale outbreak in Europe [8,31,32]. Although usually restricted to tropical areas, schistosomiasis transmission is now persisting in Corsica and the hybrid status of the parasite might have increased its invasive and adaptive capacities. Indeed, our study revealed that the European hybrids established in Corsica are highly introgressed, 77% *S*. *haematobium* origin and 23% *S*. *bovis* origin. Together with the maternal inheritance of the mitochondria this suggested that these hybrids were generated by an initial cross between a male *S*. *haematobium* and a female *S*. *bovis* with successive backcrosses with *S*. *haematobium*. This seems to confirm an ancient and mostly unidirectional introgression event(s) that could potentially be advantageous for the parasites. The hybrid status of the parasite may thus have important implication for disease control in term of host spectrum, diagnostics, and treatment in endemic areas but also Europe.

### Implications for host spectrum and parasite distribution

*S*. *haematobium* and *S*. *bovis* have different intermediate host specificities. *S haematobium* only infects snails within the genus *Bulinus* while *S*. *bovis* can also infect *Planorbarius* snails (widely present in the Iberian Peninsula), together with *Bulinus* species. The potential distribution range of the disease may thus be enhanced if the hybrid is able to infect the intermediate hosts of both parental species. Interestingly, the natural European hybrid schistosomes, that we recovered in Corsica, were not able to infect our laboratory strain of *P*. *metidjensis*, but displayed high levels of compatibility with Corsican *B*. *truncatus* (24% infection prevalence) which is consistent with previous schistosome-snail compatibility assessments for the hybrid parasite recovered directly from an infected patient in 2014 [8]. This is consistent with the predominance of *S*. *haematobium* within the genome of this hybrid, and may open the door to vector based control, e.g. by control strategies targeting these snails. However, although our findings indicate that *P*. *metidjensis* is not a host for this European hybrid we cannot exclude the possibility that other strains of *P*. *metidjensis* from Europe or Africa could be compatible and act as a host. Moreover, as the tested miracidia were collected after passage through a laboratory host, potentially inducing a population bottleneck, further testing on various hybrid field isolates is warranted. One other fundamental concern is the capacity of such introgressed schistosomes to infect livestock or other animal reservoir hosts. The zoonotic potential of the hybrids would strongly impact the parasite transmission in the field, in and out of endemic areas, and may hamper our capacity to maintain adequate control strategies as schistosomiasis treatment focuses almost exclusively on humans. Recent studies are now showing the presence of not only *S*. *bovis*, *S*. *haematobium*, and *S*. *mansoni* in rodents (hosts in which hybridization may occur), but also the occurrence of *S*. *haematobium x bovis* hybrids in such hosts in Senegal [33,34] and Benin [35] although the importance of rodents in transmission dynamics needs further exploration. Moreover, beside widespread investigation of animal reservoir in Senegal [26] to date only one study in Benin suggests that other animals such as cattle may be natural hosts for such hybrid parasites [27]. The situation in Corsica and the role of animal reservoir needs to be precisely investigated as despite ongoing transmission and developing endemicity on the island [36], no infection has been detected in livestock in the region, and the only infected animals found were two rats that do not seem to play a significant role in the transmission for this particular foci [37]. However, we cannot rule out the influence of an undetected animal reservoir such as *Ovis aries musimon*, a wild sheep native to Corsica, that have never been tested for infections [36].

### Implications for diagnostics

The hybrid status of the parasite may impair parasitological, serological and molecular diagnostics used to diagnose infections. In endemic countries, parasitological diagnosis (egg detection) is the gold standard, whereas serological tests are commonly used for imported cases of schistosomiasis in non-endemic, developed countries. In humans, schistosome eggs that are partly retained in the tissues are the cause for the disease and host-induced pathology, but are also classical tools for diagnosis and species identification. At first sight, egg morphology and their localization in the urine of infected patients in Corsica strongly suggested an *S*. *haematobium* infection [8]. Indeed *S*. *haematobium* eggs that are usually voided by the urine have a typical round to oval shape (elliptical or elongated) with a terminal spine. According to previous studies, *S*. *haematobium* eggs measure between 100–156 μm long and 40–50 μm wide with usual length between 115–135 μm long [38–40]. A previous analysis of the European hybrid eggs revealed smaller eggs (n = 15) with a mean length of 106.5 μm, a width of 42.8 μm with a spine length of 10.4 μm [32]. According to our results the eggs generally show an ovoid shape measuring 126.4 x 60.8 μm, more similar to *S*. *haematobium* eggs. This is also consistent with the introgression levels that show a predominance of S. *haematobium*-type genetic material (Table 4). However, sometimes eggs appeared intermediate with spindle or diamond shapes, which are characteristic of *S*. *bovis* eggs (usually bigger and measuring between 170–223.9 μm long and 55–66.0 μm wide) [38,41,42] (Fig 1). In addition, *S*. *bovis* eggs are released in the feces of infected animals, due to *S*. *bovis* locating around the mesenteric vessels. Thus, we could expect that hybrid parasite eggs may also be released in part in the feces of human hosts. This could explain, together with the low parasite intensity, why only 30% of patients infected in Corsica had eggs that were able to be detected in their urine [43]. The route of excretion associated with egg shape is the current standard for diagnostic and species determination, however our results confirm earlier publications showing that it is impossible to detect hybridization in schistosome species using egg morphology alone [44]. Although adult worm morphology has a limited interest for diagnostics in humans, principally because worms are not accessible, they may be useful for a taxonomic purpose. Males and females of the European hybrids were generally smaller (in length and width) when compared to both parental species [45–49], but most interestingly, the presence of spines on the tubercles of the males, a trait not found in *S*. *bovis* male, [50–52], was concordant with the predominance of *S*. *haematobium* in the genetic make up of these hybrids.

Concerning serological diagnosis, the majority of commercial tests, ELISA or IHA (indirect hemagglutination) use *S*. *mansoni* antigens. A discrepancy between those antigens and the infecting species may induce false negative results [53]. The efficiency of these commercial diagnostic kits thus needs to be reevaluated in a context of different species and hybrid forms. Finally, molecular diagnostic for urogenital schistosomiasis using PCR has already been used in urine or serum, targeting a highly repeated sequence (*Dra*I), which is restricted to the *S*. *haematobium* group of schistosomes (including both *S*. *haematobium* and *S*. *bovis*) [54,55]. We expect that this test would be efficient to detect such infection but not to identify the hybrid status of the parasite.

### Implications for treatment

Praziquantel (PZQ) is currently the main drug used to treat schistosomiasis and the application of mass chemotherapy programs is the prevailing strategy for schistosomiasis control [56]. PZQ is also efficient for treating bovine schistosomiasis, but the dose needed is quite high (60 mg/kg for 95% deworming efficacy in goats [57,58]) and cannot be considered in endemic areas where treatment capacities are primarily focused on human schistosomiasis. Moreover, it has been shown in humans that a dose of 40 mg/kg of PZQ is only 63.5% efficient for mixed infections, compared to 76.7% and 77.1%, for mono infections of *S*. *mansoni* and *S*. *haematobium*, respectively [59]. To date, neither experimental nor field trials have tested the sensitivity of hybrid parasites to PZQ. Thus, there is no current evidence that there is any difference in drug response in natural infections and changes associated to PZQ response in hybrids is still theoretical. However, a lower sensitivity to PZQ of *S*. *bovis* x *S*. *haematobium* hybrid schistosomes compared to pure *S*. *haematobium* parasites, although not tested, has been proposed to be at the origin of the spread of the hybrid forms in Senegal [60] and as discussed earlier hybridization may also affect Oxamniquine efficiency [61]. Since the genetic basis of Oxamniquine residence is known, our data suggests that OXA is not a treatment option.

### Conclusion

This work provides new insight into the *S*. *haematobium-bovis* hybrids that emerged, in Europe, revealing admixture between *S*. *haematobium* and *S*. *bovis* parasites. Such levels of genomic introgression appear to be the result of several ancient inter species crosses and subsequent backcrosses with parental species, as found in other genomic studies [11,15]. However, interestingly, it is the first time that such a high proportion of *S*. *bovis* has been identified within the genomic background of *S*. *haematobium*. As the European hybrid strain, analysed here, was originally recovered from a single infected patient infected in Corsica in 2014, it is now necessary to extend our conclusions to further infections in Corsica, and also to investigate the dynamics of these hybridization events in their original endemic areas of Africa, particularly Senegal which is thought to be the origin of the Corsican outbreak [8]. It is now also essential to precisely characterize the impact of hybridization and introgression on the parasites’ life history traits, including sensitivity to current treatments and assess the molecular mechanisms underlying these phenotypic changes. These hybrids may indeed have the capacities to dominate the geographical distribution of the parental species, but also become established is new areas fueled by ongoing climate/habitat change, together with the increased movement of people.

## Materials and methods

### Ethics statement

Housing, feeding, animal care and experiments were carried out according to the national ethical standards established in the writ of 1 February 2013 (NOR: AGRG1238753A) and accordingly to the directive 2010/63/EU of the European Parliament and of the Council of 22 September 2010 on the protection of animals used for scientific purposes. The Direction Départementale de la Cohésion Sociale et de la Protection des Populations (DDSCPP) provided the permit N°C66-136-01 to our laboratory and approved experiments on animals. The investigator possesses the certificate for animal experimentation (Decree n° 87–848 du 19 octobre 1987; authorization 007083). Samples were collected within previous studies [8]. Informed consent was collected by the InVS (National institute for Public Health Surveillance) for each patient taking part to this outbreak investigation. Serological diagnostic test conducted for each patient were part of the (standard) diagnostic work-up for schistosomiasis. Data and specimens collected were transferred anonymously to Perpignan for egg detection. The study was approved by the French Commission for Data Protection (Commission Nationale de l’Informatique et des Libertés).

### Parasite / Snail strains and experimental infections

The European *S*. *haematobium-bovis* hybrid isolate originated from eggs isolated from the urine of a locally infected tourist from Corsica and maintained in laboratory passage in Corsican *B*. *truncatus* snails and the experimental vertebrate host, the golden hamster, *Mesocricetus auratus* [8]. Briefly, eggs from the urine sample were hatched in drilling water, and the resulting larvae (miracidia and then cercariae) were used to infect intermediate host snails and subsequently laboratory hamsters. Hamsters were euthanized by intraperitonial injection of sodium pentobarbital (100 mg/kg). Adult worms were recovered from the hamsters, after portal perfusion and male/female couples were manually separated. Detailed methods are described previously [62]. *Schistosoma bovis* (isolated in 1970 in Villar de la Yegua, Salamanca, Spain) [63] and *Schistosoma haematobium* (isolated in 2015 in Barombi Kotto Lake, Cameroon) [64] were also maintained in the laboratory using *P*. *metidjensis* and *B*. *truncatus* as the intermediate hosts, respectively, and *M*. *auratus* hamsters as the definitive hosts [64]. F1 hybrids were produced after experimental cross between male *S*. *haematobium* from Cameroon and female *S*. *bovis* from Spain. Molluscs were infected with a single miracidium of the parental species to obtain male or female clonal cercariae, which were molecularly sexed as described in [64]. We simultaneously exposed hamsters to 300 cercariae of male *S*. *haematobium* and 300 cercariae of female *S*. *bovis*. Three months after infection, hamsters were euthanized, eggs were collected from the liver using a series of metal sieves (425, 180, 106, and 45 μm pore size) and F1 miracidia hatched in drilling water to infect molluscs. At patency infected snails were stimulated to produce cercariae which were used to infect hamsters (pools of 600 cercariae per hamster), with F1 hybrid adult male worms collected three months post infection.

### Hybrid parasite compatibility with snail hosts

*Planorbarius metidjensis* (n = 40) and *B*. *truncatus* (n = 40) snails were individually exposed overnight to five miracidia of the European *S*. *haematobium-bovis* hybrid strain, maintained in the laboratory, in 24-well plates containing 1ml of drilling water per well. The following morning molluscs were placed in the same tank at 26°C with a 12:12 light cycle and fed *ad libitum* for the duration of the experiment. At 35 days after infection, corresponding to the development time of the parasites in their intermediate host, snails were individually checked for parasite emission of the cercariae after light stimulation for 4 hours.

### Morphological analysis of the European *S*. *haematobium-bovis* hybrid eggs and adult worms

Morphometric analysis of *Schistosoma* eggs is a classical way to identify species. Since eggs are easily accessible in the field, excreted within stool or urine, they are commonly used to diagnose the infecting *Schistosoma* species. Twenty adult worm pairs (20 males and 20 females) were collected three months post infection after hepatic perfusion together with encysted eggs from the hamster livers. Adult worms and eggs were washed in 8.5% w/v Tris-NaCl solution for subsequent morphological analysis. Male and female worms were manually separated and stored at -80°C before subsequent DNA or RNA extraction.

After being whole-mounted on glass slides [65] the adult worms and eggs were viewed under a light microscopy and photographed using a Wild Heerbrugg M400 ZOOM Makroskop (Leica, Germany) or Dialux20 (Leitz, Germany) coupled to Nikon digital sight DS–Fi1 digital camera. All measurements were produced with ImageJ version 1.51 [66] using a real graduated scale to set up pixel numbers *vs*. mm correspondance, and drawings were done by image overlay in Adobe Photoshop CS2 version 9.0.1.

For the adult worms, the following characters were measured: worm length and width, orientation of the oral and ventral suckers, the sucker’s ratio, sucker ratio per worm length and distance from the genital opening to the anterior region. Additionally, in female worms the area of the ovary and extension of the vitellarium were measured. In males we also recorded the number of testes and the presence or absence of tegumental tubercles and characteristic tubercle spines. For eggs, the objective lens magnification was set to x10 and we measured the length (including the spine), the width (at its largest point) and the size of the terminal spine.

### DNA extraction and sequencing for *Schistosoma bovis* assembly

Two hundred clonal adult male worms of *Schistosoma bovis* from Spain were produced after monomiracidial infection of snails, sexing and hamster portal perfusion as described above. High molecular weight genomic DNA was prepared using CHEF Genomic DNA Plug Kits (BioRad). The DNA was quantified on a FEMTO Pulse, qubit and nanodrop. A total of 8.1 μg genomic DNA was used to generate a size selected PacBio library. First the DNA was sheared to an average fragment size of 45 kb by gently passing the DNA sample through a 2” long, 26 gauge needle, four times and then concentrated using Ampure PB (Pacific Biosciences 100-265-900) before the library was prepared following the standard PacBio size selected library preparation protocol using the BluePippin Size-Selection System. The library was size selected at 15kb, and run on 6 SMRT cells on the Sequel platform, generating 47.9 Gb of data.

### RNA extraction and sequencing for *Schistosoma bovis* annotation

Pools of 10–12 adult male or female worms of *Schistosoma bovis* from Spain isolated from infected hamsters were frozen with liquid nitrogen and ground using a Retsch MM400 cryobrush (2 pulses at 300Hz for 15s). Total RNA was extracted using TRIzol Thermo (Fisher Scientific) followed by DNase treatment with Turbo DNA-free kit. RNA was then purified using the RNeasy mini kit (Qiagen). The TruSeq stranded mRNA library construction kit (Illumina) was used on 300 ng of total RNA per condition. Library preparation and sequencing was performed at the McGill University in the Génome Québec Innovation Centre, Montréal, Canada on a Illumina HiSeq 4000 (100 bp paired-end reads).

### *Schistosoma bovis* genome assembly and annotation

PacBio reads were assembled using the Hierarchical Genome Assembly Process (HGAP4) de novo assembly analysis application, followed by polishing with Pilon using consensus PacBio reads and illumina short reads. Gene prediction was carried out with AUGUSTUS 3.3.1. No new training was performed for our data but the parameter set “schistosoma2” of the AUGUSTUS distribution was used. RepeatMasker 4.0.7 and RepeatScout 1.0.5 were used for repeat prediction and masking. RNAseq data from male and female adult worms was employed as external hints. This RNA-seq data was aligned with STAR version 020201. The Benchmarking Universal Single-Copy Orthologs (BUSCO) version 3.0.2 [19] was used in—genome mode and with the metazoa_odb9 dataset to assess genome assembly and annotation completeness.

### DNA extraction and sequencing for the European *S*. *haematobium-bovis* hybrid and experimental *S*. *haematobium x S*. *bovis* F1 adult worms

Genomic DNA of the European schistosome hybrid was recovered from one pool of 10 adult males and one pool of 40 adult females separately, while DNA of the experimental F1 progeny from the inter-species cross was recovered from a pool of 10 adult males. DNA was extracted using the Qiamp DNA Micro Kit tissue kit (Qiagen) followed by RNase A treatment. Genomic DNA of the European hybrid worms was then sent to Genome Quebec (https://www.genomequebec.com/) for library construction using the Illumina TruSeq kit starting from 200 ng of genomic DNA (for both males and females), and sequencing was performed on a Illumina HiSeq 2000 (100 bp paired-end reads). For the experimental F1 hybrid males, library construction was performed using the Nextera XT kit starting from 1 ng and sequenced on a Illumina NextSeq 550 (150 bp paired-end reads) on the Bio-Environment NGS platform at University of Perpignan.

### Estimation of the genomic introgression levels for the hybrid strain

The sequencing reads, with PHRED quality scores over 30, with no adapter contamination were retained for further analysis and aligned to a chimeric concatenate of the *S*. *haematobium* and *S*. *bovis* genomes using Bowtie v2.3.3.31 [67]. We used the SchistoDB *S*. *haematobium* genome v1 [17] and the *S*. *bovis* SBOS_v1.1 assembly genome produced for this study. To avoid mapping bias due to differences in assembly size between the two genomes, only scaffolds >1Mb were retained for further analyses. The genome size after concatenation of *S*. *haematobium* and *S*. *bovis* was 681.2 Mb. Mapping was thus performed by allowing each read, depending on its origin, to map against the more similar location in either the *S*. *haematobium* or *S*. *bovis* genome. We then counted the proportion of best locations of aligned reads (*S*. *bovis* or *S*. *haematobium* genomes) in the SAM files. The same procedure was applied for the mitochondrial genomes using a concatenate of the scaffold 000439F that contained the mitochondrial genomes of *S*. *bovis* (this study) and *S*. *haematobium* (GenBank accession NC_008074).

### Similarity analysis between the *S*. *haematobium* and *S*. *bovis* genomes

The two genomes were aligned using CACTUS [22]. We then processed the output, identifying all alignments blocks composed of exactly one genome part *S*. *bovis* and exactly one part *S*. *haematobium* (S1 Table). Orthologous region for SmSULT-OR (Smp_089320) was retrieved undertaking reciprocal homology searches (BlastX) against *S*. *haematobium* and the new *S*. *bovis* genome, generated as part of this study.

## Supporting information

S1 Table Global alignment summary between *Schistosoma haematobium* and *Schistosoma bovis* genomes. For each scaffold of the *S*. *haematobium* genome, the scaffold length and the number of nucleotides, which were uniquely aligned to the *S*. *bovis* genome, are reported.
(XLSX)
